# Real-life treatment patterns and time to next treatment among patients with ovarian cancer in the pre-PARP inhibitor era: the OCRWE-Finland Study

**DOI:** 10.2340/1651-226X.2024.40325

**Published:** 2024-10-16

**Authors:** Mari Lahelma, Heini Rauhamaa, Outi Isomeri, Juhana Idänpään-Heikkilä, Sari Käkelä, Nichola Roebuck, Barbara Mascialino, Sakari Hietanen, Mikko Loukovaara, Annika Auranen

**Affiliations:** a Nordic Healthcare Group, Helsinki, Finland; b GSK, Helsinki, Finland; c GSK, Brentford, Middlesex, UK; d GSK, Verona, Italy; e Department of Gynecologic Oncology, Turku University Hospital and FICAN West, Turku, Finland; f Department of Obstetrics and Gynecology and Comprehensive Cancer Center, Helsinki University Hospital and University of Helsinki, Helsinki, Finland; g Department of Obstetrics and Gynecology, Tays Cancer Centre, Tampere University Hospital and Tampere University, Tampere, Finland

**Keywords:** High-grade serous ovarian carcinoma, real-world evidence, treatment patterns, time to next treatment, progression-free survival, bevacizumab, prognostic factors

## Abstract

**Background:**

As the treatment landscape for advanced ovarian cancer (OC) evolves, it is important to understand patient outcomes in real-world clinical practice. OCRWE-Finland was an observational cohort study investigating OC outcomes, including treatment patterns, time to next treatment 1 (TTNT1), overall survival and healthcare resource utilisation, in Finland during the pre-PARPi era.

**Materials and methods:**

Patients included in OCRWE-Finland were diagnosed with OC between 2014 and 2019. Here, we report treatment patterns and TTNT1 outcomes (as a surrogate for progression-free survival) for patients in the high-grade serous ovarian carcinoma (HGSOC) cohort.

**Results:**

In OCRWE-Finland, there were 867 patients with HGSOC. Of the 811 patients who received first-line treatment, the most common regimen was surgery and adjuvant chemotherapy (53%), and 227 patients also received first-line bevacizumab. Median TTNT1 among 623 patients with stage III/IV disease was 19 months (95% confidence interval, 18–21 months), with no difference between patients with stage III or IV disease (*p* = 0.24). The presence versus absence of visible residual disease post-debulking surgery was associated with shorter TTNT1 among patients with stage III tumours (*p* = 0.031) but showed no impact for stage IV tumours (*p* = 0.55). First-line versus no first-line bevacizumab was associated with shorter TTNT1 among stages I–IV (*p* < 0.0001) but did not affect patients with stage III/IV tumours (*p* = 0.45).

**Interpretation:**

In the pre-PARPi era, prognosis for advanced OC was poor, particularly for patients with stage III tumours and visible residual disease or stage IV tumours regardless of the presence of residual disease. The increasing use of PARPis will hopefully help address the need for effective treatments in advanced OC.

## Introduction

Between 2017 and 2021, approximately 587 women per year were diagnosed with ovarian cancer (OC; including all histologies) in Finland [1]. First-line treatment for both early (stage I/II) and advanced (stage III/IV) OC typically involves surgery and chemotherapy (neoadjuvant or adjuvant, including platinum-based compounds [e.g., carboplatin] or platinum–taxane combinations [e.g., carboplatin and paclitaxel]) [2–4]. Despite optimal use of first-line surgery and chemotherapy, 70% of patients with stage III/IV OC will experience a relapse within the first 3 years [3, 5, 6]. As relapse is highly likely among patients with advanced disease, subsequent lines of treatment are imperative for most patients [7].

In advanced OC, progression-free survival (PFS) becomes increasingly short as patients progress through multiple relapses; therefore, it is paramount to extend the time from initiation of first-line treatment to the first relapse. Targeted maintenance therapies have been developed to delay disease progression or recurrence and are used either in combination with chemotherapy (continuation maintenance) or following a complete or partial response to first-line chemotherapy (switch maintenance) [7]. Based on the European Society for Medical Oncology–European Society of Gynaecological Oncology guidelines and the ICON7 trial, local Finnish guidelines recommend the use of bevacizumab for patients with stage III OC and visible residual disease after surgery (R1/R2) or those who have stage IV OC [8–10]. Bevacizumab is started with chemotherapy and is continued until disease progression, toxicity, or a treatment length of 15 months is achieved. In the real-world observational response study across eight Western countries, the use of bevacizumab concomitantly with first-line chemotherapy and as maintenance was associated with improvements in overall survival (OS) among patients with advanced OC (hazard ratio [HR] 0.62; *p* = 0.01) and a subset of patients with high-risk disease (inoperable or sub-optimally debulked stage III disease or stage IV disease; HR 0.49; *p* = 0.002) [11, 12].

The development of poly (ADP-ribose) polymerase inhibitors (PARPis) has transformed the treatment landscape for OC. The use of PARPis (niraparib and olaparib) as standard of care (SoC) treatment options has been increasing in Finland since 2018, following approval by the European Medicines Agency and subsequent national reimbursement. These agents are utilised as maintenance therapy for patients with advanced or relapsed OC who meet the reimbursement criteria, which are based on response to platinum-based chemotherapy and the presence of breast cancer gene (*BRCA*) mutations or homologous recombination deficiency [13, 14].

To our knowledge, no studies have investigated real-world treatment outcomes for patients with OC in Finland. Real-world evidence (RWE) can inform decision-making by patients, healthcare professionals, and payers, and can help contextualise results from ongoing clinical trials. Although PFS is a valuable alternative to OS in clinical trials, it is difficult to measure in real-world studies [15–17]. Time to next treatment (TTNT) is an established, robust, and objective real-world endpoint that is used as a surrogate for PFS; TTNT encompasses duration of disease control, treatment tolerability, and patient adherence to treatment [18, 19].

The aim of the OCRWE-Finland study was to obtain real-world data from the pre-PARPi era on treatment patterns, TTNT, OS, and healthcare resource utilisation (HCRU) using data from patients diagnosed with OC in Finland between 2014 and 2019. During this period, the SoC for OC according to Finnish treatment guidelines was first-line chemotherapy and bevacizumab maintenance therapy for high-risk patients. In this article, we report findings on treatment patterns and TTNT for patients included in the high-grade serous OC (HGSOC) cohort of OCRWE-Finland. Although we report outcomes for all disease stages, we focus more on patients with advanced (stage III/IV) HGSOC. The OS outcomes can be found in the corresponding article by Mari Lahelma et al. [20]. Results on HCRU will be published elsewhere.

## Methods

### An overview of OCRWE-Finland study design

OCRWE-Finland was a multicentre, retrospective, observational cohort study based on secondary use of hospital medical records in Finland. The study involved the OC patient population from the three largest University Hospitals in Finland (Helsinki University Hospital [HUS], Turku University Hospital [VSSHP], and Tampere University Hospital [PSHP]), which treat approximately 50% of patients with OC in Finland. Treatment of OC is largely centralised to University Hospitals, reflecting the SoC in Finland. Patients were followed from the baseline period (≤ 3 months prior to the date of diagnosis) until the end of the study period.

This study included adult female patients (≥18 years of age) who were diagnosed with OC (including ovarian, fallopian tube, or primary peritoneal cancer) between 1 January 2014 and 31 December 2019 and whose home municipality was in the HUS, VSSHP, or PSHP areas. Potentially eligible patients were identified by diagnosis codes (International Classification of Diseases 10th Revision [ICD-10]; HUS: C48, C56, C57.0; PSHP: C56, C57.0, C57.8; VSSHP: C48, C56, C57.0) from hospital records/databases at the participating centres. Patients were excluded if they had participated in a clinical trial between 1 January 2014 and 31 December 2019.

The primary objective of the study was to describe TTNT (defined in the ‘Analysis of TTNT and treatment patterns’ section) for patients with OC and in a cohort of patients with HGSOC. Secondary study objectives are reported in the Supplementary Material.

### Analysis of TTNT and treatment patterns

Here, we report findings for the primary objective of TTNT (including analyses to identify prognostic factors for TTNT) and treatment patterns as a secondary objective. This report focuses on HGSOC, the most prevalent histology among patients in OCRWE-Finland.

Time to next treatment 1 (TTNT1) was defined as the time between the beginning of first-line chemotherapy and the beginning of second-line treatment, date of death, or loss to follow-up through to the end of the study period (31 December 2019); patients who were lost to follow-up were censored at the end of the study period. TTNT was also analysed by use of bevacizumab in the first-line setting; this analysis did not consider the length of bevacizumab use.

Assessment of treatment patterns and patient demographics/characteristics is described in the Supplementary Material and Supplement Table S1.

### Data management and statistical analysis

Data were extracted from patients’ medical records and hospital databases within the study sites. To ensure patient anonymity, only results for which there were a minimum of five patients with available data are reported. For descriptive statistics, data are presented as mean (standard deviation) for continuous variables and number (%) for categorical variables. Kaplan–Meier survival analysis was used to estimate the probability of TTNT1, and a Cox proportional hazards model was applied to identify prognostic factors for TTNT1, through univariate and multivariate analyses. Each explanatory variable was first assessed through univariate analysis; subsequently, significant variables were introduced to the multivariate analysis to identify independent prognostic factors for TTNT1. Prior to analysis, the proportional hazard assumption was tested for covariates based on scaled Schoenfeld residuals and graphical diagnostics. Analysis of demographics/characteristics for patients who received prior bevacizumab versus those who did not (including subgroups by advanced disease stage and presence of residual disease post-debulking surgery) was performed by Chi-squared test for categorical variables and unpaired Student’s *t*-test for continuous variables. The *p*-values of < 0.05 were considered statistically significant.

Additional details on data management/statistical analysis are provided in the Supplementary Material.

## Results

### Patient characteristics

In total, 867 patients were included in the HGSOC cohort (51% of the total OCRWE-Finland population). A breakdown of the HGSOC cohort by disease stage is shown in Supplementary Material, Supplement Figure S1, and patient age at first diagnosis by residual tumour status is shown in Supplementary Material, Supplement Figure S2.

### Treatment patterns in the first-, second-, and third-line settings

**Figure 1:**
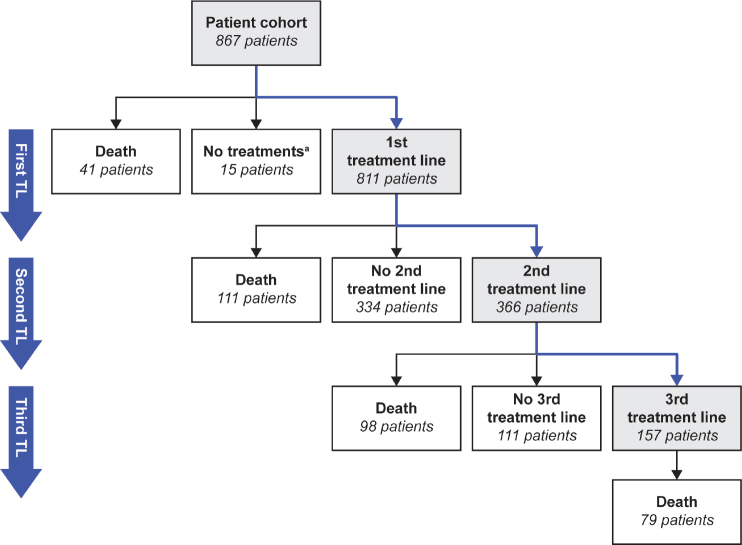
Treatment line progression. ^a^Includes a few patients with an irregular first treatment line. TL: treatment line.

**Figure 2:**
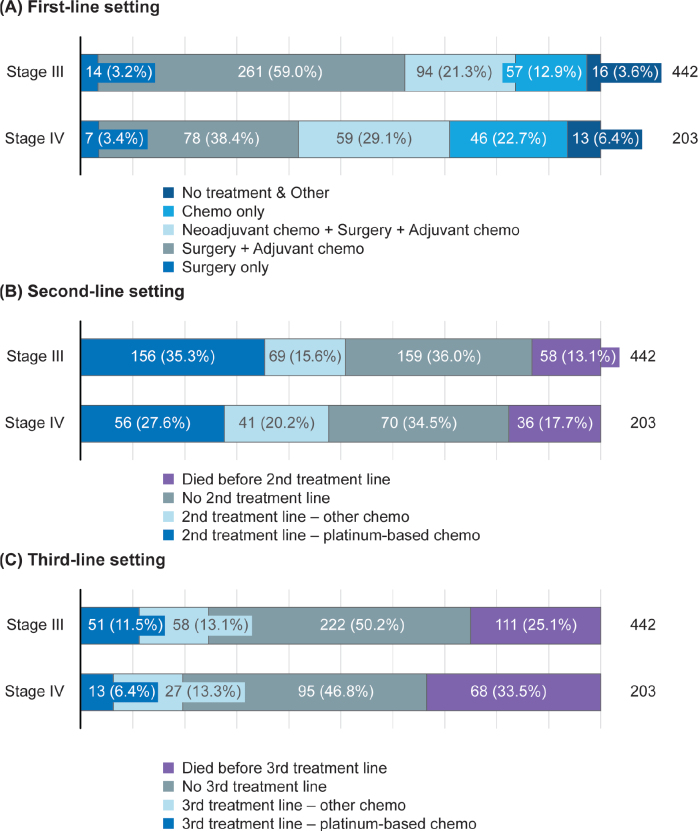
Treatment patterns in patients with stage III/IV disease in the (A) first-line, (B) second-line, and (C) third-line settings. Chemo: chemotherapy.

Treatment line progression is shown in Figure 1. In the first-line setting, 456 (53%) patients received surgery and adjuvant chemotherapy. For stage I/II disease, surgery and adjuvant chemotherapy was the most common first-line regimen. In stage III/IV disease, there was wide variation in regimens used (Figure 2A). Among all disease stages, a total of 765 patients received chemotherapy as part of their first treatment line, of whom almost all received platinum-based regimens (n = 760; 99%). The most common chemotherapies, often administered in combination, were carboplatin (*n* = 756; 99%), paclitaxel (*n* = 629; 82%), docetaxel (*n* = 155; 20%) and doxorubicin (*n* = 55; 7%). Paclitaxel plus carboplatin was the most common first-line chemotherapy combination (*n* = 628; 82%). The median number of treatment cycles was six (interquartile range [IQR]: 5–8) and the median duration of treatment was 117 days (IQR: 105–174).

Approximately 24% of patients with stage I/II disease received a second line of platinum-based or other chemotherapy during the study period. Approximately 50% of patients with stage III/IV disease received second-line treatment during the study period, and the most common regimen was platinum-based chemotherapy (Figure 2B). Approximately 23% of patients with stage III/IV disease received third-line treatment during the study period, and this was typically a non-platinum-based regimen (Figure 2C).

Of the 811 patients who received first-line treatment, approximately 38% of patients received bevacizumab in at least one treatment line. The characteristics of the 227 (28%) patients who received bevacizumab in the first-line setting are presented in Table 1. Seventy-nine (10%) patients received bevacizumab in the second-line setting, of whom 45 (57%) received bevacizumab in combination with platinum-based chemotherapy and 34 (43%) received bevacizumab in combination with non-platinum-based chemotherapy (Table 2).

**Table 1:** Demographics and characteristics of patients who received bevacizumab in the first-line setting (*n* = 811).

Characteristic	No bevacizumab in first-line setting (*n* = 584)	Bevacizumab in first-line setting (*n* = 227)	*p* value
Age (years), mean (SD)	69.1 (10.4)	65.3 (10.0)	**< 0.001**
BMI, *n* (%) < 18.5 kg/m^2^ 18.5–24.9 kg/m^2^ 25–29.9 kg/m^2^ 30–34.9 kg/m^2^ 35–39.9 kg/m^2^ > 39.9 kg/m^2^ Missing	12 (2.1) 213 (36.5) 206 (35.3) 67 (11.5) 32 (5.5) 15 (2.6) 39 (6.7)	6 (2.6) 95 (41.9) 67 (29.5) 31 (13.7) 5 (2.2) 11 (4.8) 12 (5.3)	0.071
Location, *n* (%) Fallopian tubes Ovaries Peritoneum and retroperitoneum Uterine ligaments, adnexa, others	74 (12.7) 392 (67.1) 71 (12.2) 47 (8.0)	18 (7.9) 161 (70.9) 38 (16.7) 10 (4.4)	**0.026**
Stage, *n* (%) I II III IV Missing	85 (14.6) 43 (7.4) 299 (51.2) 107 (18.3) 50 (8.6)	> 5 < 5 127 (56.0) 83 (36.6) 8 (3.5)	**< 0.001**
Residual tumour, *n* (%) R0 R1 R2 Missing	235 (40.2) 78 (13.4) 46 (7.9) 225 (38.5)	35 (15.4) 63 (27.8) 61 (26.9) 68 (30.0)	**< 0.001**
Risk level, *n* (%) Stage III R0 Stage III R1/R2 Stage IV R0 Stage IV R1/R2 Missing	128 (21.9) 87 (14.9) 27 (4.6) 29 (5.0) 313 (53.6)	10 (4.4) 78 (34.4) 20 (8.8) 42 (18.5) 77 (33.9)	**< 0.001**
*BRCA1/2*, *n* (%) No mutation carrier Mutation carrier Unknown Missing	132 (22.6) 16 (2.7) 17 (2.9) 419 (71.7)	77 (33.9) 12 (5.3) 15 (6.6) 123 (54.2)	0.494

Only patients with first-line treatment (*n* = 811) were included in the analysis. BMI: body mass index; SD: standard deviation.

**Table 2:** Use of bevacizumab in at least one line of treatment (*n* = 306).

Number of patients		Treatment line in which bevacizumab was first received		
		Bevacizumab in first-line setting	Bevacizumab in second-line setting	Bevacizumab in third-line setting
Further lines of treatment with bevacizumab	Bevacizumab in first-line setting	215	–	–
	Bevacizumab in second-line setting	11	62	–
	Bevacizumab in third-line setting	< 5	> 5	11

A total of 505 patients did not receive bevacizumab in any treatment line and data relating to bevacizumab use were missing in 56 patients. Table shows the number of patients who received bevacizumab in each treatment line; one treatment of bevacizumab was defined by combining all doses that were given within 59 days from one another.

An overview of the use of first-line bevacizumab across different risk groups is shown in Table 1. Among patients with stage III tumours and no visible residual disease post-debulking surgery (R0; low-risk group; *n* = 138), 10 patients (7%) received bevacizumab in the first-line setting, and they were significantly younger than those who had not received bevacizumab in the first-line setting (mean age 55.2 years *vs*. 66.3 years; *p* = 0.002). Among patients with stage III tumours and visible residual disease post-debulking surgery (R1/R2; high-risk group; *n* = 165), 78 patients (47%) received bevacizumab in the first-line setting; factors significantly associated with the use of first-line bevacizumab in these patients included age, body mass index (BMI), and residual tumour status (Supplement Table S2). Among patients with stage IV R0 disease (high-risk group; *n* = 47), 20 patients (43%) received bevacizumab in the first-line setting; there were no significant characteristic differences between patients who had or had not received first-line bevacizumab (all *p* > 0.05). Among patients with stage IV R1/R2 disease (high-risk group; *n* = 71), 42 patients (59%) received first-line bevacizumab and they were significantly younger than those who had not (mean age 66.8 years *vs.* 71.4 years; *p* = 0.046).

### TTNT1

**Figure 3:**
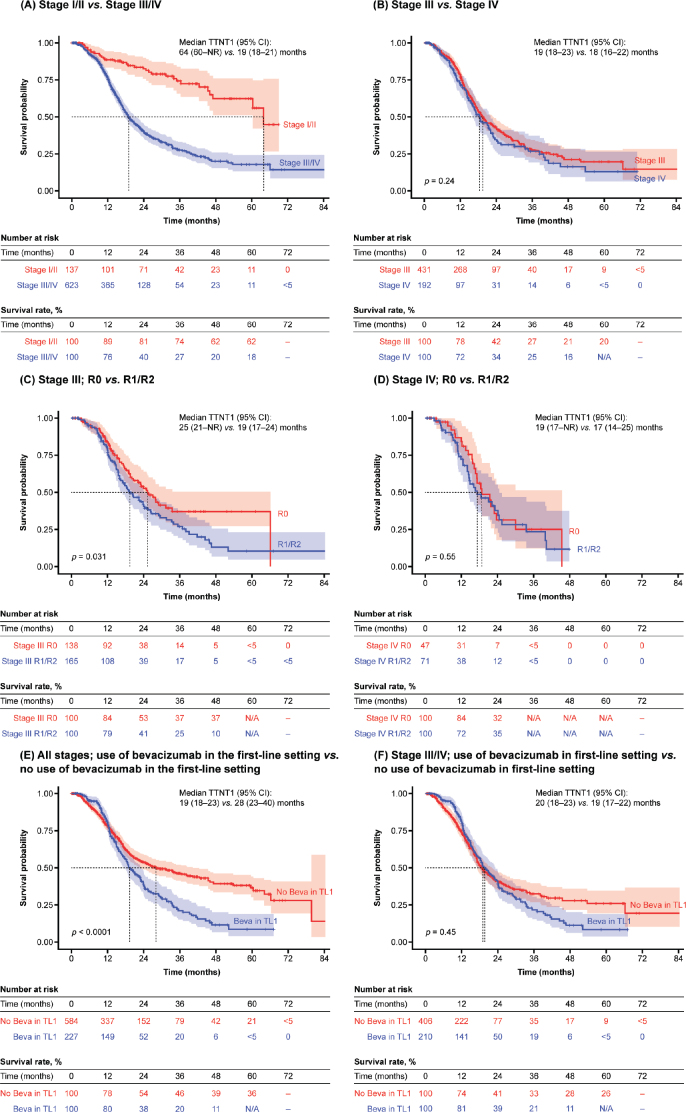
TTNT1 by stage of disease, presence or absence of visible residual disease status post-debulking surgery, and first-line bevacizumab use. Beva: bevacizumab; CI: confidence interval; NA: not available; NR: not reached; TL1: treatment line 1; TTNT1: time to next treatment 1.

Median TTNT1 was 64 months (95% confidence interval [CI]: 60–not reached [NR]) in patients with stage I/II disease and 19 months (95% CI: 18–21) in patients with stage III/IV disease (Figure 3A). Patients with stage IV disease had similar TTNT1 (median: 18 months; 95% CI: 16–22) compared with patients with stage III disease (median: 19 months; 95% CI: 18–23; *p* = 0.24; Figure 3B). Among patients with stage III disease, TTNT1 was significantly shorter for those with visible residual disease post-debulking surgery versus those without (median: 19 months [95% CI: 17–24] and 25 months [95% CI: 21–NR], respectively; *p* = 0.031; Figure 3C). However, TTNT1 was similar for patients with stage IV disease, regardless of the presence of residual disease (median: 17 months [95% CI: 14–25] and 19 months [95% CI: 17–NR]; *p* = 0.55; Figure 3D).

Across all disease stages, despite a slight added benefit in the first 12 months, patients who received bevacizumab in the first-line setting had a significantly shorter TTNT1 than those who had not (median: 19 months [95% CI: 18–23] *vs*. 28 months [95% CI: 23–40], respectively; *p* < 0.0001; Figure 3E). However, among patients with stage III/IV disease only, TTNT1 was similar, regardless of first-line bevacizumab use (median: 20 months [95% CI: 18–23] and 19 months [95% CI: 17–22], respectively; *p* = 0.45; Figure 3F).

In univariate analysis of TTNT1, younger age, stage III at initial diagnosis, stage IV at initial diagnosis, presence of residual tumour post-debulking surgery, use of neoadjuvant chemotherapy (NACT), use of bevacizumab in the first-line setting and peritoneal origin were associated with significantly shorter TTNT1 (all *p* < 0.05; Table 3). In multivariate analysis of TTNT1, younger age, stage III at initial diagnosis, stage IV at initial diagnosis, the presence of residual tumour, and use of NACT were identified as independent prognostic factors (all *p* < 0.05; Table 4), while first-line bevacizumab use was not independently associated with TTNT1 (HR 1.84; 95% CI: 0.61–1.15; *p* = 0.272).

**Table 3:** Univariate analysis for TTNT1 (*n* = 867).^a^

Characteristic	Patients	HR	95% CI	*p*-value
Age (years), mean (SD)	68.6 (10.8)	0.99	0.98–1.00	**0.049**
Location Fallopian tubes Ovaries Peritoneum and retroperitoneum Uterine ligaments, adnexa, others	95 586 119 67	– 0.99 1.52 1.27	– 0.71–1.38 1.03–2.24 0.76–2.12	– 0.961 **0.037** 0.354
Stage I II III IV	92 45 442 203	– 0.86 3.38 3.90	– 0.41–1.81 2.20–5.21 2.47–6.16	– 0.692 **< 0.001** **< 0.001**
Residual tumour R0 R1 R2	270 141 107	– 1.73 2.15	– 1.28–2.32 1.55–2.98	– **< 0.001** **< 0.001**
BMI < 18.5 kg/m^2^ 18.5–24.9 kg/m^2^ 25–29.9 kg/m^2^ 30–34.9 kg/m^2^ 35–39.9 kg/m^2^ > 39.9 kg/m^2^	18 327 285 102 40 27	– 1.36 1.61 1.57 1.66 1.93	– 0.56–3.32 0.66–3.93 0.63–3.95 0.61–4.46 0.69–5.43	– 0.499 0.300 0.336 0.319 0.211
*BRCA1/2* No mutation carrier Mutation carrier Tested	212 28 32	– 0.94 0.88	– 0.53–1.64 0.53–1.48	– 0.816 0.637
NACT No Yes	704 163	– 1.40	– 1.11–1.77	**0.004**
Bevacizumab in TL1 No Yes	584 227	– 1.55	– 1.25–1.91	**< 0.001**

a Only complete cases were used for each analysis. BMI: body mass index; CI: confidence interval; HR: hazard ratio; NACT: neoadjuvant chemotherapy; SD: standard deviation; TL1: treatment line 1; TTNT1: time to next treatment 1.

P-value <0.05 was considered to be statistically significant.

**Table 4:** Multivariate analysis for TTNT1 (*n* = 867).^a^

Characteristic	Patients	HR	95% CI	*p*-value
Age (years), mean (SD)	67.6 (10.1)	0.98	0.96–0.99	**0.001**
Location Fallopian tubes Ovaries Peritoneum and retroperitoneum Uterine ligaments and adnexa, and others	53 373 55 27	– 0.68 0.76 0.64	– 0.45–1.03 0.44–1.30 0.32–1.27	– 0.071 0.318 0.200
Stage I II III IV	57 30 303 118	– 1.44 2.09 2.66	– 0.63–3.29 1.20–3.62 1.44–4.89	– 0.381 **0.009** **0.002**
Residual tumour R0 R1 R2	264 139 105	– 1.53 2.40	– 1.08–2.17 1.61–3.59	– **0.016** **< 0.001**
NACT No Yes	375 133	– 1.46	– 1.08–1.97	– **0.013**
Bevacizumab in TL1 No Yes	351 157	– 1.84	– 0.61–1.15	– 0.272

a Only complete cases were used for each analysis. CI: confidence interval; HR: hazard ratio; NACT: neoadjuvant chemotherapy; SD: standard deviation; TL1: treatment line 1; TTNT1: time to next treatment 1.

P-value <0.05 was considered to be statistically significant.

## Discussion

We analysed TTNT1 as a primary objective and treatment patterns as a secondary objective using real-world data for patients with HGSOC treated in routine clinical practice in Finland between 2014 and 2019. Across all disease stages, surgery and adjuvant chemotherapy was the most common first-line treatment regimen, although there was wide variation in first-line treatment regimens among patients with stage III/IV disease. Of note, a large proportion of patients with stage III/IV disease did not initiate second-line treatment (approximately 50%). While 15% of these patients had died before reaching the second-line setting, we speculate that the remaining patients did not receive subsequent treatment, perhaps due to censoring prior to switching treatment, patients being transferred to a different hospital to receive treatment (and so being lost to follow-up), or patient reluctance to initiate further chemotherapy due to poor tolerability. Furthermore, patients diagnosed close to the end of the follow-up period of the study (31 December 2019) may not have been followed up for sufficient time to detect disease progression and switching treatment. In the second- and third-line settings, the most common treatment regimens for stage III/IV disease were platinum-based chemotherapy and non-platinum-based chemotherapy, respectively.

In stage I/II disease, we observed a median TTNT1 of 64 months. Although the earlier diagnosis of these patients implies better OS than later stages [21], this finding highlights that these patients remain at risk of relapse, even after 5 years. It remains unclear whether closer monitoring of these patients, over many years following their diagnosis, would improve their outcomes [22]. It is important to acknowledge that the stage I/II TTNT1 survival rate after 48 months (62%) was based on a low number of patients at risk, so these findings should be interpreted with caution and verified through further RWE studies.

In stage III/IV disease, we observed a median TTNT1 of 19 months. A similar number of patients with stage III versus stage IV disease received second-line chemotherapy (51% *vs*. 48%, respectively). Interestingly, while the OS outcomes from this study demonstrated statistically significantly improved OS for patients with stage III disease compared with stage IV disease [20], no difference in TTNT1 was observed between these groups. This suggests that while both patient groups experience disease progression and switch to a second-line treatment at a similar time, patients with stage III disease survive longer following progression than those with stage IV disease.

The SoC for OC is primary surgery, aiming to reduce tumour burden and minimise residual disease [23]. Among patients with stage III tumours, we observed that the presence of visible residual disease post-debulking surgery was associated with a shorter TTNT1 compared with no residual disease (median: 19 months *vs*. 25 months, respectively). TTNT1 was the same for patients with stage III tumours and visible residual disease and those with stage IV tumours and no visible residual disease post-debulking surgery (median: 19 months for both). Among patients with stage IV tumours, the presence of visible residual disease post-debulking surgery had no impact on TTNT1 (median: R1/R2 17 months *vs*. R0 19 months, respectively). In Finland, most patients with stage IV disease are candidates for surgery if there is a chance of achieving R0. Our findings suggest that patients with stage IV disease will likely still have a poor prognosis even if R0 is achieved with surgery. However, as these subgroups comprise relatively few patients, these findings should be interpreted with caution.

Approximately 24% of patients with stage III/IV disease received NACT plus surgery and adjuvant chemotherapy in the first-line setting. NACT is typically used for patients if complete debulking surgery is unlikely to be successful (e.g., those with extra-abdominal disease) [23]. In this study, while multivariate analysis demonstrated a significant impact of NACT on TTNT1, further analysis of this impact was limited by the dataset. The authors anticipate that the ongoing re-run of this study will provide further insight into the impact of NACT on TTNT and whether the percentage of patients treated with NACT in Finland has increased since this study was conducted, due to changes related to the coronavirus disease 2019 (COVID-19) pandemic (as seen in Italy [24]).

We observed that most patients who received first-line bevacizumab had either stage III R1/R2 disease or stage IV disease, in line with Finnish treatment guidelines [8]. However, we also observed that 10 patients with stage III R0 disease received first-line bevacizumab. Across all disease stages, there was an initial treatment effect in the first 12 months, and the use of first-line bevacizumab was associated with significantly shorter TTNT1 versus patients who had not received first-line bevacizumab. In patients with stage III/IV disease, first-line bevacizumab provided a treatment effect in the first 12 months, no apparent effect in the second year of treatment, and no statistically significant effect on TTNT1 overall. The multivariate analysis showed that advanced disease stage, younger age, presence of residual tumour, and use of NACT were independently associated with shorter TTNT1, but first-line bevacizumab was not statistically significant (HR 1.84; *p* = 0.272).

The decrease in TTNT1 (including those with stage III/IV disease only) could be because most patients receiving first-line bevacizumab were at higher risk of disease progression. Likewise, the group of patients who did not receive first-line bevacizumab represented those with lower risk of disease progression; for example, as per local Finnish guidelines, most patients with R0 disease after surgery did not receive bevacizumab. Our results could also be explained by a rebound effect observed previously with bevacizumab in serous OC that is characterised by increased disease progression after around 1 year of treatment (as was observed in our study) and following the cessation of bevacizumab treatment [25]. For the 10 patients with stage III R0 disease who received first-line bevacizumab, we speculate they may have been younger patients who were offered a more aggressive treatment strategy to optimise their outcomes and prevent recurrence, or patients with other high-risk tumour characteristics. This observation may also reflect different prescribing policies across institutions. Confirmation of why these patients may have been considered to require an aggressive treatment strategy was not collected in this real-world study. Together, our observations indicate that there remains an unmet need for novel treatment options in addition to bevacizumab for patients at high risk of disease progression. The re-run of this study will provide insight into whether the introduction and increasing use of PARPis as maintenance treatment for patients with OC in Finland is helping to address this unmet need.

As few patients with HGSOC in OCRWE-Finland were tested for *BRCA* mutations (27.7%), it is unsurprising that *BRCA* status was not significantly associated with TTNT1. In this population, testing of *BRCA* mutations was introduced in clinical practice only after PARPis received reimbursement in Finland. This is aligned with a 2020 retrospective cohort study of the real-world CancerLinQ database; of 2654 patients with OC, only 600 patients (23%) had been tested for a *BRCA1/2* mutation, of whom 17% were positive [26]. Historically, the prognostic value of *BRCA* mutations has been unclear. Some studies have demonstrated an association between improved PFS and OS outcomes in patients with *BRCA* mutations, whereas others have observed no effect [27].

Overall, this analysis provided valuable insights regarding pre-pandemic treatment patterns and TTNT1 outcomes based on long-term RWE from patients with OC treated in Finland during the pre-PARPi era, representing historic clinical practice when the only available maintenance treatment was bevacizumab. During this time, prognosis for patients with advanced OC was poor, particularly for those with stage III tumours and visible residual disease or stage IV tumours irrespective of the presence of visible residual disease post-debulking surgery. For patients with stage I/II disease, the risk of progression remained possible, even after 5 years. This suggests a high unmet need for effective treatment options in this patient population. This is supported by a real-world analysis by the Ovarian Real-World International Consortium from 2012–2018; among 3344 patients with epithelial OC, at least 21% of patients treated with systemic anticancer therapy had recurrent disease after second-line treatment and some patients received up to 11 lines of systemic treatment [28].

Looking forward, the study re-run will shed light on the impact of the COVID-19 pandemic and increasing PARPi use on treatment outcomes and confirm whether the advent of targeted therapies is helping to improve prognosis in patients with OC. An optimal treatment algorithm would allow patients to have access to all effective treatment options. To ensure patients receive the highest SoC, a multidisciplinary team should oversee their management and apply individualised treatment strategies using a precision medicine approach based on patients’ characteristics, molecular biomarkers, and sensitivity to chemotherapy.

### Study’s limitations

Retrospective studies are limited by the completeness and quality of medical records and reliability of data abstraction. However, this design was most appropriate for collecting data on clinical practice during this rapidly progressing treatment era. To address any potential abstraction errors, source data verification was used. Poor coverage of *BRCA* mutation status may also limit the interpretation of these findings. Furthermore, data on the use of secondary cytoreductive surgery were not available within the study dataset. The OCRWE-Finland population reflects the three largest University Hospitals in Finland (approximately 50% of patients with OC in Finland) but may not be representative of smaller hospitals.

Data on real-world PFS were not available in our dataset, and, thus, we were unable to examine any disparities between TTNT and real-world PFS. Based on the literature, using TTNT as a proxy for PFS may result in overestimation or underestimation of real-world PFS [16, 18]. Reasons for this may be that patients have already experienced disease progression at the time of changing treatment (perhaps due to a delay in identifying a suitable subsequent treatment option [18]) or may switch treatment for reasons other than progression (e.g., tolerability, patient choice, or physician decision). However, real-world PFS is difficult to accurately capture in routine clinical practice, as it is not typically assessed as regularly or consistently as in clinical trials. During the research period, local Finnish guidelines recommended patient follow-up at the symptom clinic every 3 months during the first year, every 4 months during the second year, every 6 months during the third year, and then yearly follow-ups for the fourth and fifth years. Further analysis of the specific timing of initiation of subsequent treatment lines for patients in this study (e.g., during scheduled follow-up visits or in-between visits) would have provided further insights regarding the value of TTNT compared with real-world PFS (e.g., whether TTNT may be a better indicator of recurrence than PFS) and the effectiveness of real-world follow-up protocols in Finland; these data were unavailable within the dataset and should be investigated in future research. However, a recent retrospective real-world analysis of 362 patients with OC treated with PARPis as first-line maintenance in the United States between 2017 and 2022 observed a strong correlation between TTNT and real-world PFS, regardless of *BRCA* mutation status, supporting the use of TTNT as a proxy for PFS in studies of OC [29].

## Conclusion

This is the first real-world study to report treatment patterns and TTNT1 outcomes for patients in Finland who were diagnosed with HGSOC during the pre-PARPi era. During this time, prognosis was poor for patients with advanced disease, particularly those with stage III tumours and visible residual disease post-debulking surgery and those with stage IV disease. Our findings highlight an unmet need for effective treatment options in addition to bevacizumab for these patients, which is hopefully being addressed by the increasing use of PARPis.

## Supplementary Material

Real-life treatment patterns and time to next treatment among patients with ovarian cancer in the pre-PARP inhibitor era: the OCRWE-Finland Study

## Data Availability

Information on GSK’s data-sharing commitments and requesting access to anonymised individual participant data and associated documents from GSK-sponsored studies can be found at www.clinicalstudydatarequest.com.
