# Supplementary material for: Real-life treatment patterns and time to next treatment among patients with ovarian cancer in the pre-PARP inhibitor era: the OCRWE-Finland Study
Source: Acta Oncol. 2024 Oct 16;63:40325. doi: 10.2340/1651-226X.2024.40325 (PMC11495144; doi:10.2340/1651-226X.2024.40325)
Supplement: Real-life treatment patterns and time to next treatment among patients with ovarian cancer in the pre-PARP inhibitor era: the OCRWE-Finland Study [file AO-63-40325-s1.pdf]

Supplementary material has been published as submitted. It has not been copyedited, or typeset by Acta Oncologica

Supplementary Methods

Study design

The target sample size for this study was approximately 550–700 patients from each site. This number was determined sufficient for key descriptive analyses to be representative of the sample population as a whole, while considering the feasibility of patient inclusion. The study complied with the ethical principles of the Declaration of Helsinki and the requirements of the European Union General Data Protection Regulation. Patient informed consent was not required, as the study was conducted under the Act on the Secondary Use of Health and Social Data.

Study endpoints

Secondary endpoints of the study included ovarian cancer (OC)-related healthcare resource use, baseline patient demographics and disease characteristics, treatment pathways, summary measures of disease progression, response to treatment, time to first and subsequent disease progressions and overall survival (OS) in patients with OC in the overall population and those with high-grade serous OC (HGSOC). Exploratory analyses were performed to identify any associations between type of treatment and OS, between time to next treatment (TTNT) and OS, and between key characteristics and OS.

Treatment pattern assessments

For treatment pattern assessment, patients were grouped into categories for each line of treatment; maintenance therapies and anti-angiogenic treatments were excluded from treatment line classifications (**Supplement Table S1**). All drugs (except bevacizumab) received within 49 days of one another were grouped together as first-line treatment. The use of bevacizumab was defined by identifying patients with records of bevacizumab use; if bevacizumab administration was identified within the start and end of the active treatment line, the line was classified to have included bevacizumab. One treatment of bevacizumab was defined by combining all doses of the drug that were given within 59 days of one another. When the patient received their last dose of chemotherapy, the treatment line ended even if the patient was still receiving bevacizumab. Recurrence was defined as 2 weeks prior to the start of new treatment.

Study assessments

Patient demographics and characteristics assessed in the study included age, body mass index (BMI), anatomical location of tumour at initial diagnosis, histological classification, disease stage, *BRCA* genetic status, and visible residual tumour after surgery.

BMI was calculated based on structured data on weight and height. The first measure (maximum of 3 years prior to diagnosis) for each patient was included in the analysis.

Initial stage of OC was determined based on International Federation of Gynaecology and Obstetrics (FIGO) staging classification: stage I (I, IA, IB, IC), stage II (II, IIA, IIB), stage III (III, IIIA, IIIB), stage IV (IV, IVA, IVB), and unknown (no data suggestive of FIGO stage). FIGO stage was determined based on text mining of electric health records for patients from Helsinki University Hospital (HUS) and Tampere University Hospital (PSHP). Stage classification of patients from Turku University Hospital (VSSHP) was available in the data (classified by clinician). Expressions related to stage followed by Roman numeral (I–IV) with or without letter (A–C) were looked for with pattern matching. If more than one stage was reported for the patients, the earliest was selected.

Classification of residual disease was based on reported R classification or residual tumour classification by the surgeon for patients undergoing surgery in the first-line setting: R0 (complete resection of visible disease), R1 (largest residual tumour size ≤1 cm, or small volume of residual tumour), R2 (largest residual tumour >1 cm, or large volume of residual tumour), unknown (no information suggesting presence or absence of residual tumour for operated patient). The patient's first record for residual tumour in the data was included in the analyses. If more than one value was reported, the highest value was included. Residual disease was determined based on electronic health records for patients from HUS and PSHP. Classification of patients from VSSHP was available in the data (classified by clinician). Data were searched by both pattern matching and manually through patient records.

BRCA mutation status was classified as follows: mutated *BRCA* (diagnosis clearly indicated an expression of pathological mutation in either *BRCA1* or *BRCA2*), wild-type *BRCA* (diagnosis clearly indicated no expression of pathological mutations or variations in *BRCA1* or *BRCA2*), or unknown (no data related to *BRCA1/2* test results were found). *BRCA* status was based on text mining of pathological examinations and diagnoses for patients from HUS. Classification of patients from PSHP was available in the data (classified by clinician). No data were obtained from VSSHP.

Patients who did not receive first-line treatment were excluded from the TTNT analysis.

Minimisation of bias

In this study, selection bias was avoided by analysing the total population in the three University Hospitals participating in this study. Recall bias and transfer bias were mitigated by using secondary data from reliable hospital databases and patients' medical records. Confounding bias was minimised by stratification of the study population. Citation bias was minimised by employing a multidisciplinary research team in the study.

Data management and statistical analysis

Patient data were collected, combined, and provided in pseudonymised form by Findata, the permit holder of the study. Findata operates under the Secondary Data Act, disclosing information in such a way that the data protection of the individual is maximised [1]. Patients were identified by a unique study code to preserve confidentiality. Standard imputation was applied for missing days and/or months; for other missing data, only available data were analysed. All analyses were performed using RStudio, R version 4.1.0 (R Core Team 2021, R Foundation for Statistical Computing).

Supplement Table S1. Treatment pattern assessment

First-line treatment	Second- and third-line treatment
Surgery only, surgery plus adjuvant chemotherapy, NACT plus surgery plus adjuvant chemotherapy, chemotherapy only, and other. Other included patients who did not receive treatment and those whose treatment did not match the other categories (e.g., patients with platinum resistance before surgery and patients with only NACT + surgery) NACT and adjuvant chemotherapy were defined as chemotherapy received within 120 days of primary surgery Chemotherapy in this setting included both platinum- and nonplatinum-containing regimens: carboplatin, paclitaxel, gemcitabine, doxorubicin, docetaxel, topotecan, etoposide, cisplatin, oxaliplatin, and vinorelbine	Platinum-based chemotherapy and other chemotherapy

NACT: neoadjuvant chemotherapy.

Supplement Table S2. Patient demographics and characteristics among patients with stage III R1/R2 disease post-debulking surgery (high-risk group) who received bevacizumab in the first-line setting

**Characteristic**	**No bevacizumab in first-line setting (*n* = 87)**	**Bevacizumab in first-line setting (*n* = 78)**	***p* value**
**Age (years), mean (SD)**	71.6 (7.5)	65.6 (9.4)	**<0.001**
**BMI, *n* (%)**			
<18.5 kg/m^2	<5	<5	**0.006**
18.5–24.9 kg/m^2	30 (34.5)	35 (44.9)	
25–29.9 kg/m^2	34 (39.1)	23 (29.5)	
30.0–34.9 kg/m^2	8 (9.2)	13 (16.7)	
35–39.9 kg/m^2	>5	0 (0)	
>39.9 kg/m^2	0 (0)	<5	
Missing	<5	<5	
**Location, *n* (%)**			
Fallopian tubes	9 (10.3)	>5	0.406
Ovaries	59 (67.8)	59 (75.6)	
Peritoneum and retroperitoneum	14 (16.1)	10 (12.8)	
Uterine ligaments, adnexa, others	5 (5.7)	<5	
**Residual tumour, *n* (%)**			
R0	0 (0)	0 (0)	**0.012**
R1	58 (66.7)	36 (46.2)	
R2	29 (33.3)	42 (53.8)	
***BRCA1/2*, *n* (%)**			
No mutation carrier	29 (33.3)	27 (34.6)	0.058
Mutation carrier	<5	<5	
Unknown	<5	>5	
Missing	48 (55.2)	46 (59.0)	

BMI: body mass index; SD: standard deviation.

Supplement Figure S1. Staging distribution among all included patients (*n* = 867)

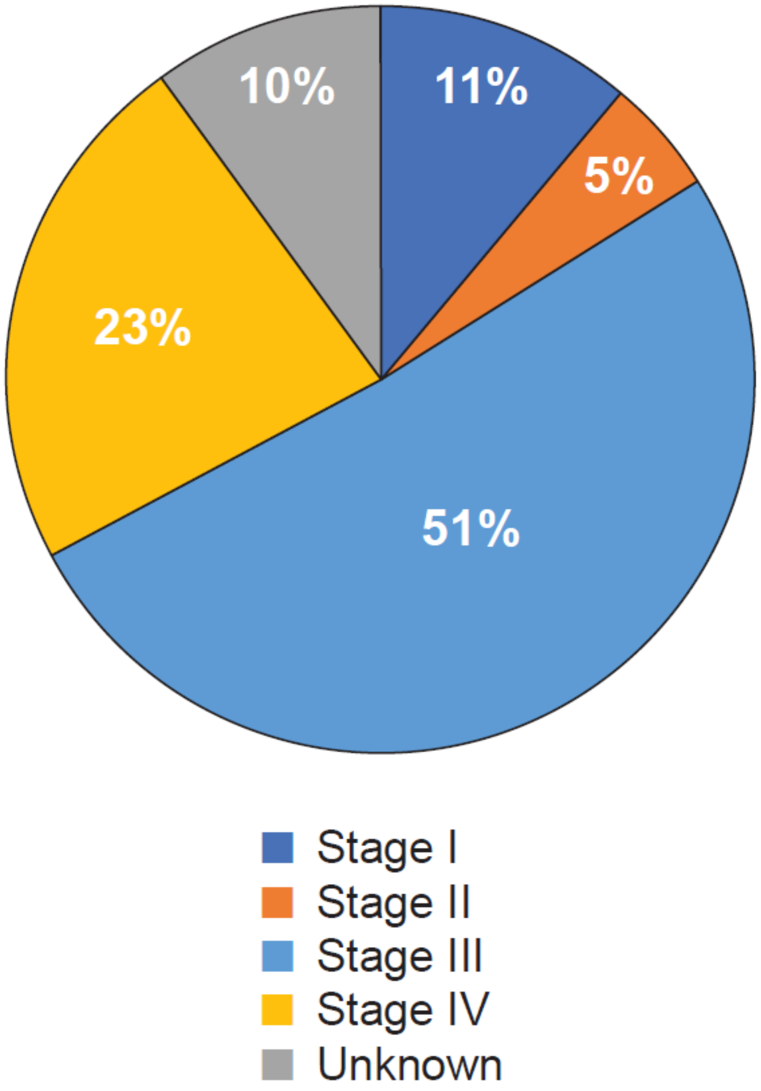

Supplement Figure S2. Age at the first diagnosis of OC by residual tumour status

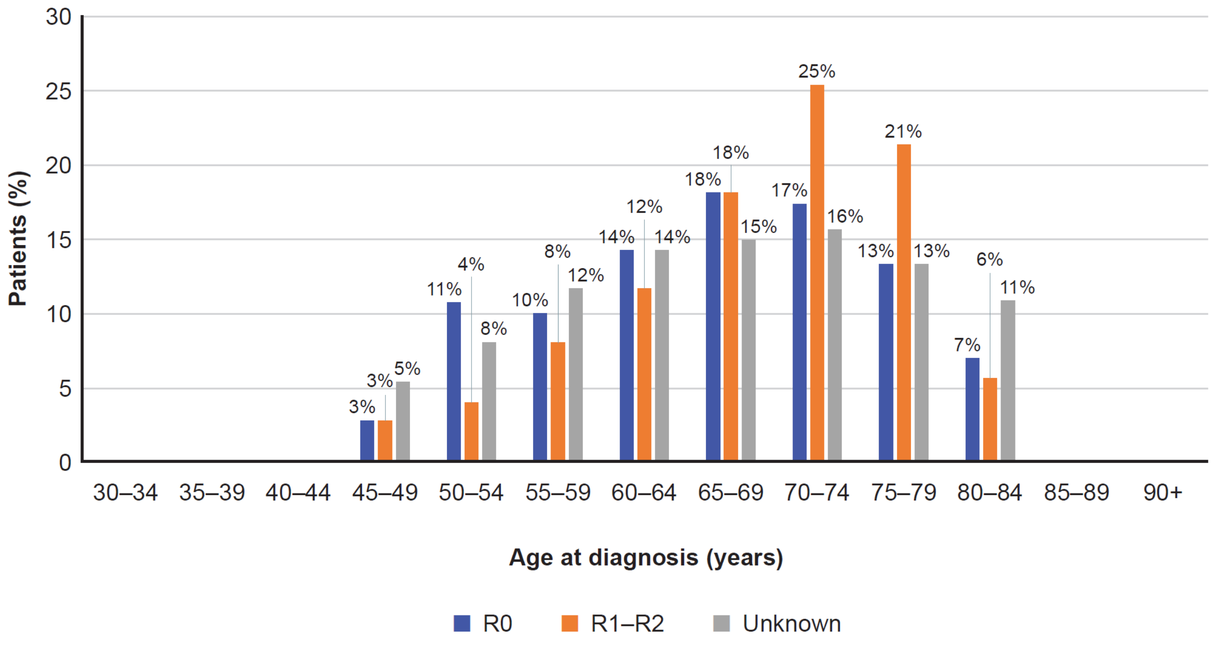

OC: ovarian cancer.

References

1. FINDATA: Social and Health Data Permit Authority. Data 2023 [updated 23 March 2023; cited 2023 14 April]. Available from: https://findata.fi/en/data/.
